# Building Transnational Bodies: Norway and the International Development of Laboratory Animal Science, ca. 1956–1980

**DOI:** 10.1017/s026988971400009x

**Published:** 2014-06

**Authors:** Tone Druglitrø, Robert G. W. Kirk

**Affiliations:** University of Oslo tone.druglitro@tik.uio.no; University of Manchester robert.g.kirk@manchester.ac.uk

## Abstract

This article adopts a historical perspective to examine the development of Laboratory Animal Science and Medicine, an auxiliary field which formed to facilitate the work of the biomedical sciences by systematically improving laboratory animal production, provision, and maintenance in the post Second World War period. We investigate how Laboratory Animal Science and Medicine co-developed at the local level (responding to national needs and concerns) yet was simultaneously transnational in orientation (responding to the scientific need that knowledge, practices, objects and animals circulate freely). Adapting the work of Tsing (2004), we argue that national differences provided the creative “friction” that helped drive the formation of Laboratory Animal Science and Medicine as a transnational endeavor. Our analysis engages with the themes of this special issue by focusing on the development of Laboratory Animal Science and Medicine in Norway, which both informed wider transnational developments and was formed by them. We show that Laboratory Animal Science and Medicine can only be properly understood from a spatial perspective; whilst it developed and was structured through national “centers,” its orientation was transnational necessitating international networks through which knowledge, practice, technologies, and animals circulated.

More and better laboratory animals are today required than ever before, and this demand will continue to rise if it is to keep pace with the quickening tempo of biological and veterinary research. The provision of this living experimental material is no longer a local problem; local, that is, to the research institute. It has become a national concern, and, in some of its aspects . . . even international. (William Lane-Petter 1957, 240)

Writing in 1957, William Lane-Petter, a leading British expert on laboratory animal production, provision, and management, identified three formative and interrelated demands that had shaped and sustained the rapid growth of the biomedical sciences during the years immediately following the Second World War: more laboratory animals, better quality laboratory animals, and the growing importance of coordinating responses to these demands at the international level. Lane-Petter identified laboratory animals as a necessary resource for the continued progress and success of the biomedical sciences. Good science required good tools; the quality of research relied on the quality and availability of research tools. The creation of Laboratory Animal Science and Medicine as an auxiliary biomedical discipline in the postwar period, a process in which Lane-Petter played a pivotal role, was a response to these concerns (Kirk 2010). Despite arising independently and distinctly at the level of the nation state, characteristic of the laboratory animal problem was the belief that adequate answers could be found only at the international level. In this article we explore how Norwegian national concerns were both informed by, and informed, the international endeavor to co-coordinate a transnational response to questions of laboratory animal production, provision, and management. In doing so we reveal how laboratory animal science and medicine was mutually constituted at the level of national practice and international activity, mobilizing discourses on scientific standards to produce a transnational ideal grounded in material cultures of the laboratory.

Historical studies have shown that sourcing adequate experimental animals (Clarke 1987; Kirk 2008; Druglitrø 2012), together with their means of production and standardization (Clause 1993; Rader 2004), have long been central to the success of the biomedical sciences. Analytically, historical scholarship has principally focused on explaining the co-development of highly specific experimental organisms within highly specific research trajectories at the local level (Kohler 1994; Clarke and Fujimura 1992; Ramsden 2011). In contrast, scholars from a wide range of other disciplines, including science studies, cultural studies, and geography, emphasize the international outlook of the biomedical sciences (Jasanoff 2005; Sunder Rajan 2006) and fundamentally spatial aspects of laboratory animal production and use (Davies 2010, 2012a, 2012b). These different perspectives are shaped not only by disciplinary preferences but also by periodization. Historical literature tends to focus on early- to mid-twentieth century chronology, whereas later literature prioritizes late twentieth century to the near present. By focusing on the development of laboratory animal science and medicine from the mid- to late-twentieth century, this article places these two literatures in closer dialogue, whilst contributing to the development of transnationally orientated histories of science (van der Vleuten 2008; Sivasundaram 2010), as well as the study of standardization as a driver of the internationalization of science (Drori et al. 2003; Lampland and Leigh Star 2009).

Practices of laboratory animal production and provision thus form part of the little studied auxiliary infrastructure that has sustained the phenomenal growth of the biomedical sciences in the latter half of the twentieth century. Laboratory animal science and medicine was (and is) principally an auxiliary specialty, existing to facilitate biomedical research proper (though it has and does conduct original research to this end). Importantly, this meant that the work of laboratory animal science was one step away from defined biomedical research agendas, operating at a generic level.^1^ Systems for producing and procuring experimental animals have always been a necessary component of biomedical research. However, prior to the formation of laboratory animal science and medicine in the immediate decades following the close of the Second World War, these practices predominantly consisted of ad hoc local arrangements, based on tacit and unverified knowledge that co-developed with research agendas in dispersed laboratories. In contrast, laboratory animal science and medicine sought to create a new specialist discipline located in particular buildings alongside the laboratory, wherein varied biomedical expertise, including genetics, pathology, microbiology, epidemiology, nutrition, and veterinary medicine, were mobilized to secure the provision of affordable high quality laboratory animals to all regardless of geographic locality. Laboratory animal science and medicine was also distinct in that the new expert knowledge and technical practices created under its name, though produced locally, were international in orientation. The purpose was to provide the biomedical sciences with highly specified species with known histories, as opposed to the ad hoc and often critically unknown animals that had previously been the experimental norm (cf. Kirk 2008). We argue that the differences between national practices, systematized through international relations, came to serve as the medium through which transnational standards of laboratory animal production, provision, and management were created. The ultimate aim was to reconfigure laboratory animals *as* transnational bodies. As such, from its beginning laboratory animal science was consciously engaged in the construction of shared languages and practices, as well as the networks and infrastructures through which science travels, as a means to provide the tools by which the biomedical sciences were given form and function.

The article engages with the aims of this special issue by adopting a historical approach to study the role of Norway in the development of laboratory animal science in the immediate decades following the close of the Second World War. We examine how national difference operated as both necessary and productive factors, mobilized at the international level so as to create and establish transnational standards for laboratory animal science, and also reconfigure the animal itself as a transnational body. We begin by outlining our use of the term “transnational” and its relation to the terms “national” and “international.” We then examine the stuttering national movement to establish a center for laboratory animal science in Norway during the 1950s, which failed in 1958 only to be revived to partial success in the early 1960s having gained traction from the mobilizing international developments. In the third section we investigate further how national differences informed, and were informed by, the construction of international infrastructures that were designed to facilitate the formation of laboratory animal science as a new and critically important auxiliary biomedical field (focusing particularly on how Norwegian laboratory animal science influenced and operated through these emerging international networks). Finally, we examine a specific case study, being the creation of Specific Pathogen Free laboratory animals, illustrating how international activities were orientated toward creating transnational standards and, ultimately, building transnational bodies.

## National Centers, International Infrastructures, and Transnational Standards

In their seminal study of the relationship of science, standardization, and globalization, Drori and his colleagues demonstrate how “expanded science encourages the institutionalization of national procedures of data collection and sets such practices in the manner most prevalent in the international arena” (Drori et al. 2003, 287). Initially emerging in response to biomedical scientists attempting, with varying degrees of success, to enroll the nation state as a means to constitute a regulatory infrastructure for the production and provision of laboratory animals, laboratory animal science followed this schema. In 1947, for instance, the British Medical Research Council established the Laboratory Animal Bureau (later Centre) to improve the “present haphazard system, or lack of system” by acting as a “clearing house for information on the supply and demand for experimental animals” (Kirk 2010, 63).^2^

The formation of laboratory animal science can only be understood from a perspective that recognizes its origins and activities to have been always already dispersed. Despite the initial focus on national infrastructures for standardizing and regulating laboratory animal production, the actual standards so produced were international in outlook. Lane-Petter, for instance, for whom there was no comparable national center for laboratory animals when he first began the work of establishing British standards, nevertheless toured the world in order to gather information on local practices, research institutes, visiting universities, pharmaceutical laboratories, commercial breeding establishments, and other places of scientific interest (Kirk 2010). Laboratory animal science was premised on science and technology being on the move, its role was to facilitate the circulation of techniques and technologies. These techniques, therefore, were imagined as transnational even though they originated at the national level and traveled through international networks and infrastructures. We examine how “transnational” characteristics of laboratory animal science were actively (as opposed to passively) enacted in a specific locality, Norway, so as to shape public health politics, the material cultures of the Norwegian biomedical sciences. Norway was a special case, both in terms of representing central positions in the International Council for Laboratory Animals (ICLA) and because they were particularly dependent on international collaboration to establish a national laboratory animal industry.^3^ “Transnationality” was thus in our view enacted as part of the Norwegian processes, and not something that existed outside of it, and in that way worked to change the practices of Norwegian biomedical sciences and public health politics (cf. Asdal 2012). For this reason, we also suggest that laboratory animal science emerged simultaneously at the national and international level. In 1955 the Council for International Organizations of Medical Sciences (CIOMS) became interested in the laboratory animal problem.^4^ The same year the International Union of Biological Sciences (IUBS) appointed a committee to examine laboratory animal provision on an “international scale” (IUBS 1955, 117–8). By 1956, UNESCO, working with CIOMS, IUBS, and representatives of the then existing national laboratory animal organizations in the UK, France, and the USA, agreed on a roadmap for the development of “international co-operation in the field of laboratory animals.”^5^ The vehicle for international co-operation was the International Committee on Laboratory Animals (ICLA), established in 1956 under the auspices of IUBS and CIOMS, sponsored by UNESCO, and with the Swedish zoologist Sven Otto Hörstadius acting as Chairman and Lane-Petter as Executive Secretary. ICLA was the principle international infrastructure linking the geographically dispersed “centers” of laboratory animal science, serving to facilitate the circulation of knowledge, practice, and animals. The purpose of ICLA was to catalyze and co-ordinate the international production, provision, and utilization of laboratory animals about transnational standards, creating “high quality” laboratory animals and making them available to all laboratories regardless of local circumstance.

“Transnational” was not a term that would have been used or recognized by those who worked to establish laboratory animal science. Nor was the word “global” often invoked, though a comparable meaning was conveyed by the use of “world.”^6^ Indeed, ICLA came into being as a result of successfully manufacturing “the urgent need for an international body entrusted with the study of laboratory animals problems as a whole and on a world basis.”^7^ Nonetheless, we employ the term transnational to capture the extent to which laboratory animal science emerged dynamically, mutually constituted by local needs and the desire to work across local limitations so as to facilitate the free circulation of knowledge. Consequently, we develop transnationality as an analytic concept that captures more than “cross-border flows” pointing to something that “transcends” borders (van der Leuten 2008, 978), where the relative movement would be from one static and identifiable space to another, where actors, objects, and cultures on each side of the borders remain fixed (Mitchell 1997, 103). We wish to privilege the extent to which the national and transnational co-develop over time. Thus, we presuppose that national and transnational concerns always shaped local practice.^8^ Our aim is to avoid framing aspects of scientific culture, politics, and practice within a national and transnational dichotomy. Instead, we seek to articulate their mutual, interrelated development. To do so, we adapt the concept of “friction,” being “the awkward, unequal, unstable, and creative qualities of interconnection across difference,” which Anna Tsing advances as a means to capture the idea of global universals being “not as truths or lies but as sticky engagements” (Tsing 2005, 5–6). Such engagements are always embedded within local, material contingencies, yet remain transnational and mobile. If globalization is movement across space, friction is the force that both limits movement and makes it possible. It is both productive (enabling actions) and limiting (curtailing the same). The tension between the two is what drives creative action. Such was laboratory animal science, facilitating and shaping the improvement of laboratory animal production and provision simultaneously at the level of local material practice and transnational scientific standards.^9^ However, we avoid the term globalization in favor of transnational as the developments we trace were not global in their agenda. Laboratory animal science was focused only on the spaces where animal-dependent science functioned, which, though geographically dispersed and infinitely varied, were assumed, ideally, to operate by regimes that transcended their locality.

The goal of laboratory animal science was to establish transnational standards of laboratory animal production, provision, and maintenance. In practice, we argue that this ideal took the form of materially reconfiguring the laboratory animal so as to serve as a transnational body made for and of the laboratory (itself conceived as a transnational space). Progress toward transnational standards of laboratory animal science gained traction through the mobilization and systematization of national difference. Thus, transnational agreements on laboratory animal science whether conceptual (such as nomenclature) or material (such as the animal body) emerged through the building of international infrastructure. Foremost was ICLA, however, their work was frequently initiated at the level of local concerns, emanating from national centers of (sometimes self-proclaimed) expertise in laboratory animals. Thus, ICLA was simultaneously a product of numerous national events that created a demand for transnational standards as well as an instigator of such demands where they had yet to form. Indeed, as in the case of Norway, the existence of ICLA contributed to establishing a need for the systematic development of laboratory animal science even in cases where the national biomedical sciences had failed to do so.

## National Centers: The Norwegian Case

Norway lacked an established biomedical research community sufficiently powerful to compel centralized state intervention in laboratory animal provision as occurred in the UK, USA, and elsewhere. Nevertheless, worries were expressed by the research community that they were not able to meet emerging public health challenges due to the inconsistent quality and availability of laboratory animals. In 1953 the pathologist Olav Torgersen and biologist Ernst Wulff Rasmussen (of the University of Oslo) warned the Norwegian National Research Council (NRC) that:

the need for laboratory animals is increasing, particularly those of rats and mice, and for several institutions the situation is precarious … researchers have this spring been “out of work” for weeks and months due to the shortage of animals, and last year we had to get animals from different breeders, which resulted in a serious epizootic outbreak that almost spoiled all of the long-term research projects.^10^

Concerns primarily focused on the quantity of animals available, which was insufficient, particularly in view of the expected expansion of diagnostic work and vaccine testing and production due to the state-sponsored vaccination programs. Between 1953 and 1958 a working party at the NRC headed by Olav Torgersen developed various proposals for the national organization of laboratory animal provision that were presented to the Health Directorate placed under the Ministry of Social Affairs (*Sosialdepartementet*) for the establishment of a large-scale provision unit for laboratory animals to be placed in the vicinities of the largest institutions in Oslo using animals.^11^ The proposed breeding station was said to solve the challenges of public health, as it would democratize the practices of biomedicine both in the sense of making animals available to all scientific institutions, and as tools for ensuring public health.^12^

The NRC plan for a breeding station was in line with official politics on public health work. The Director of the Norwegian Board of Health from 1938 to 1972, Karl Evang, worked to integrate the biomedical sciences, particularly microbiology, virology, and epidemiology, into clinical therapy (Thue & Helsvig 2011).^13^ Evang sought to adapt Anglo-American systems of integrated biomedical healthcare, which he had witnessed during the Second World War, to Norway.^14^ This healthcare model prioritized scientific administration and laboratory informed medicine in the belief that scientific medicine was the route to national health (Sturdy and Cooter 1998). Evang was convinced that close cooperation between the political administrative system working to develop national health politics and medical expertise was the best way to practice and perform preventative medicine, which would ensure the making of a “welfare state” in which people could enjoy “social and economic well-being” (Berg 2002, 121; Slagstad 2001, 364–365). Facing the threat of international epidemics such as polio, small pox, and the flu in the postwar era, collaboration between laboratory-based medicine (including diagnostics and vaccine production) and the political administration was regarded as crucial in order to manage the spread on a national scale.^15^ Within this system, laboratory animals served not only as research tools but also as components of routine diagnostic tests and, increasingly, as instruments for the development and testing of new vaccines and drugs. Laboratory animals thus grew in importance as the biomedical sciences became ever more integrated within Norwegian political health initiatives. Ironically, despite their best efforts, Torgersen and the working party failed to convince the Health Directorate that their plans for a centralized breeding station was a feasible and economically sound project.^16^ To the Health Directorate the construction of a large-scale breeding unit for laboratory animals seemed like an exaggerated move, as its cost was not obviously justified by the current needs or by the level of activity in the biomedical community. The NRC, which had been supporting the work of the working party, concluded that if the Health Directorate did not support the project, the expense of any scheme outweighed the gains.^17^

Failing to sway the government, early Norwegian pioneers of laboratory animal science looked beyond the nation state to bolster their cause. The international development of laboratory animal science could be mobilized to provide new impetus, and a more compelling argument, for state involvement in the organization of national infrastructures for the production, provision, and maintenance of laboratory animals. This was a not a new strategy: After World War II, Norwegian officials were increasingly involved in developing systems and regulations for handling issues concerning energy, environment, and laboratory animals. This work has been important for ensuring a simultaneous growth *and* protection of national industry and nature (see Asdal 2011).

By the early 1960s access to high quality animals, for research as well as routine diagnostic use, was increasingly recognized by the Norwegian state to be an intrinsic consideration in the wider “fight against epidemics” and consequently a central component in ensuring the health and safety of the Norwegian people (Lerche 1962). The link between national health and medical sciences had been established already at the turn of the nineteenth century (Asdal 2008); however, the scale of the laboratories, the quantity of animals needed, and the importance of international collaboration due to the transnational character of the diseases (for instance polio), rose to new prominence in the postwar era compared to fifty years earlier.^18^ A leading proponent of experimental medicine was Christian Lerche, virologist and director of the National Institute for Public Health, who in 1960 initiated a second wave of agitation for state involvement in the systematization of laboratory animal provision in Norway. Supported by colleagues such as Helge Stormorken (Norwegian School of Veterinary Science), together with visits from Lane-Petter representing the recently formed ICLA (ICLA 1961, 4), the argument for provision was strengthened through the claim that Norway “needed” to develop laboratory animal science if it was to be included in the emerging international biomedical science community.^19^ Without critical information on the general health, age, pathogenic and genetic history, Norwegian scientists, it was claimed, would struggle to get their work published in reputable international journals (Lerche 1962). Helge Stormorken, a veterinarian, research scientist, and expert in bleeding diseases, wrote to the NRC to press for formal Norwegian participation at ICLA, explaining: “Country after country has witnessed an emerging crisis in providing adequate animal resources to meet the growing demands of the larger scientific research institutions. In our country, we have recently arrived at this point.”^20^

Accordingly, it would be foolhardy not to learn from ICLA and in doing so enable Norwegian biomedical scientists to participate in the emerging international biomedical community. In this way the existence of ICLA was mobilized to support the development of laboratory science at the national level. The “quality” of Norwegian science was now framed as dependent upon the “quality” of its laboratory animals. To stand outside international processes of standardizing the practices of laboratory animal science and medicine was to risk isolation and most importantly fail to advance science and ensure public health. Participation, on the other hand, would sustain the credibility of Norwegian science on the world stage. In principle, Norwegian biomedical research could contribute on an equal footing with that of any other nation, providing it possessed an auxiliary infrastructure of laboratory animal science that met with agreed international standards of quality. In 1960 Lerche became the first representative of Norway to join ICLA, a move which was intended to catalyze the establishment of a formal “Laboratory Animal Centre for Norway” (ICLA 1961, 11).^21^

## National Frictions and the International Infrastructure of Laboratory Animal Science

From ICLA’s perspective, facilitating the systematization of laboratory animal production and provision in Norway was part of a wider agenda to build an international infrastructure for laboratory animal science operating through transnational standards of practice that would work toward “improving the quality and supply of laboratory animals” (Lane-Petter 1958, 58). Quality was less a defined property than a transcendent ideal determined by local circumstance:

quality is not a general characteristic, but refers to special properties, of the animals, such as genetic constitution, freedom from pathogenic infections, nutritional status, and other things. For some types of work, for example cancer research, genetic constitution is of paramount importance; for others, such as long term experiments or immunological research, freedom from infection is more important; while for still others, a high degree of Uniformity of specific responses matters most of all. (Lane-Petter 1957, 61)

As such, the notion of quality operated to bridge the ICLA ideal of transnational standards whilst meeting the local demands of specific laboratory cultures, research trajectories, and experimental practices. Nevertheless, these different meanings of quality required definition and interpretation in order that they could be accurately communicated: this was to be the work of ICLA.

Even the definitions of inbred strains of mice, which had been subjected to intense genetic standardization for a number of decades (Rader 2004), were not all they were assumed to be. In 1957, Lane-Petter reported that contrary to expectation there was “great confusion, [as] different sublines bear the same name, or closely related sublines carry totally unrelated names” (Lane Petter 1957, 61). Any discussion of quality was premised on the certainty that a mouse strain known as C3H would be the same in any laboratory across the world, thus a standard nomenclature for laboratory animals became a necessary pre-requisite for all of ICLA’s work. Accordingly, with financial support from UNESCO, M. A. Sabourdy, Director of the French Centre de Sélection des Animaux de Laboratoire, was tasked with developing a nomenclature that would become the agreed transnational language for defining laboratory animals. However, the process of establishing definitions and a standard nomenclature was far from “top down.” On the contrary, standardized nomenclature emerged from an extended process of negotiation between ICLA members and local users of laboratory animals worldwide. Between 1958 and 1962, Sabourdy spent much of his time visiting laboratories across the world observing their animals, collecting information and soliciting views and opinions. After publication, the agreed terms continued to be subject to prolonged negotiation and amendment (ICLA 1964, i-v). In this way, a laboratory animal nomenclature emerged through sustained negotiation and interaction between transnational and local actors. In his initial report, Sabourdy acknowledged this interactive character of the process, as well as the contested status of some terms, by placing each definition in one of three categories, non-controversial, slightly controversial and strongly controversial (Sabourdy 1962). This illustrates not only how ICLA’s work operated through close interaction with local scientific material cultures and practices, but also the extent to which such interaction produced the friction necessary to allow for productive traction and thus progress in developing a transnational nomenclature for laboratory animals.

Much of ICLA’s work toward improving the supply of laboratory animals was structured around developing standards to improve local breeding techniques according to principles of efficiency and reliability. The aim was to guarantee the sustainable production of laboratory animals making animals available as and when required. In many ways, improving laboratory animal supply was, in fact, indivisible from that of quality, particularly regarding the question of animal health (see more on this, below). However, several aspects of laboratory supply, not least the question of efficiently moving animals from sites of production to sites of use, raised practical challenges that could only be addressed at the international level. In this respect, different species of laboratory animals posed different challenges. Some, such as mice, guinea-pigs, and rats were commercially produced, others, including cats, dogs, and common agricultural animals, tended to be procured locally as required, whereas still others, particularly the various species of nonhuman primates, were caught wild in their natural habitats. Systematizing the distinct challenges posed by these various routes for sourcing animals, as well as developing species-specific regimes for the shipment of animals to safeguard their health and welfare, required ICLA to consider how transnational standards could coalesce with national laws (controlling animal transport across borders) as well as numerous local practices for animal shipment employed by commercial couriers.^22^ Here, ICLA’s international structure facilitated the development of agreed standards for the transportation of animals across diverse political, economic, and geographic stakeholders. This work was critically important for building an international infrastructure around which a commercial laboratory animal industry might grow before consolidating into global multinationals by the late twentieth century (Kirk 2005).

Structurally, membership in ICLA was open only to national members nominated by their relevant government. Where respective national centers for laboratory animals had been established, it was conventional for their directors to serve also as national representatives at ICLA. In countries that lacked national centers, an appropriate person was nominated, usually whoever was considered to be the leading local expert in laboratory animals. This international framework structured much of ICLA’s early work, particularly its attempt to reconfigure laboratory animals as a global resource. It also helped to balance the universal ideal with the pragmatic needs of local circumstance. As Lane-Petter acknowledged:

The needs of research workers are the same in any country. … But the extent to which those needs are met in each country varies, often considerably. It depends on the number and distribution of universities and research institutes; the development of pharmaceutical research and industry, which are the biggest numerical consumers of animals; the presence of commercial suppliers of animals; the country’s geography, which often governs the pattern of supply; and the financial and other resources available for effecting improvements. It is undoubtedly true to say that the poorer a country’s resources for research, the less can it afford to waste them on inferior materials such as bad animals, and the greater the need, therefore, to improve these materials; and the same applies to individual laboratories. From this it follows that the countries less advanced in their research are most in need of help, and it also follows that there is a need in every country. (Lane-Petter 1957, 68)

One of the first tasks, therefore, was to map existing local geographies as a first step toward developing strategies for shaping these to meet ICLA’s transnational agenda. In 1956, with the partial exception of the UK, the USA, and France, where respective national centers had begun the work of identifying and mapping the hitherto local ad hoc practices of sourcing laboratory animals, there was little information available to know what animals were currently used in biomedical research, what they were used for, or where they were sourced from. Accordingly, ICLA initiated a program of laboratory animal surveys across all UNESCO member states known to be conducting biomedical research. Each survey was conducted by a national representative who issued standard forms to all animal-dependent laboratories soliciting information on sources and types of animals used, problems encountered, and future expectations. To supplement this quantitative picture, Lane-Petter identified and visited many of the largest users to conduct informal qualitative enquiries. By 1959 surveys had been completed in twenty countries, including Australia, Czechoslovakia, Denmark, Finland, Norway, West Germany, Iceland, Italy, Japan, Poland, Sweden, Switzerland, and Turkey, published in two volumes under the title International Survey of the Supply, Quality and Use of Laboratory Animals (ICLA 1959).

Significantly, the Nordic national surveys, covering Denmark, Finland, Iceland, Norway, and Sweden, were undertaken collectively by Onther F. Bahr of Karolinska Institute, Sweden (ICLA 1957, 3). Throughout the 1950s ICLA treated these countries as one, making the Nordic region what could be termed a “bordered” transnational space. One reason for this was that the biomedical sciences were comparatively modest in each nation state. This, combined with shared strong historical and cultural affinities, had in any case driven a collective Nordic identity for the development of science and technology (Stråth 2006, 133).^23^ Nevertheless, an effect of these surveys was to catalyze interest in the laboratory animal problem in nations where laboratory animal science was yet to formalize as a field of expertise and identity.^24^ Norway was no exception. In 1963, the Department of Experimental Animal Medicine and Laboratory Animal Supply at the National Institute of Public Health (NIPH) was established as a first step toward a promised Norwegian center. Stian Erichsen, a Norwegian veterinarian who had been working in the animal house of the newly established Department of Virology at the NIPH, was appointed director. By this point the NIPH had become widely known as Norway’s “primary weapon” in the promotion of public health, leading the fight against infectious disease, particularly tuberculosis and polio.^25^ Laboratory animals, as has already been stated, were critical to this work. For Erichsen, laboratory animal science provided a means to integrate veterinary expertise into this wider mobilization of biomedicine in the service of Norwegian healthcare. He quickly established himself as a leading expert in laboratory animal science, becoming the first formally appointed “Laboratory Animal Science Veterinarian” in Norway.^26^ However, Erichsen was equally committed to the development of laboratory animal science as a transnational expertise. Having inherited Lerche’s role as Norwegian representative at ICLA, Erichsen was appointed to the governing board in 1963. In 1967 he succeeded Lane-Petter as Secretary-General. Thereafter, the international hub of laboratory animal science shifted from the UK to Norway. Erichsen’s entitlement as the head of ICLA was far from given, and in many ways even unforeseen regarding Norway’s size and significance, or lack thereof, in biomedical sciences internationally. As pointed out above, Erichsen’s position in international development was a strategic move for the Norwegian biomedical community, but it was also a strategic move for ICLA, as it would be less controversial for a person representing a country of small-scale research than a representative from a country where biomedical sciences were shaped by a range of strong stakeholders such as industry, animal protection societies and scientists.

Erichsen effectively ran ICLA from 1967 to 1979, a period in which the organization established laboratory animal science as a transnational endeavor. Equally as significant, in this period ICLA worked to shape the transnational governance of animal experimentation. Erichsen co-ordinated the scientific response to the (then) European Communities’ desire to establish European regulations for animal experimentation, and was active in communicating the practical aspects of laboratory animal science, working to formalize Lane-Petter’s practice of visiting countries as a WHO supported program of “Visiting Experts” in laboratory animal science.^27^ ICLA expert members acted as ambassadors for laboratory animal science, visiting any nation that sought advice on the organization of laboratory provision. In 1971 and 1973, for instance, Erichsen visited Thailand providing advice on the design, layout, equipping, and fitting of a Thai National Laboratory Animals Center. This was to serve as a place for training in laboratory animal science as well as for producing high quality laboratory animals. The work of building the Center, which began in 1976, posed several challenges. Not least was the temperature. Western animal houses were pushing the boundaries of air conditioning technology for maintaining consistent temperature and hygienically secure environments. In Thai summer, when temperature climbed to 40 Celsius in the summer, air conditioning struggled to maintain the temperature in the low thirties. Here, Erichsen employed his principle of “standardization wherever possible,” acknowledging the need to mediate between local needs and transnational standards (Erichsen 1961b). In this case the only detrimental effect was found to be a marked decline in reproduction rates. Other unique considerations included close attention to vermin prevention, particularly mosquitoes. The National Laboratory Animals Center was completed in 1979, producing its first animals in 1980 (National Laboratory Animal Center of Thailand 2002), subsequently becoming an important hub for the development of laboratory animal science in South East Asia.

In these ways, ICLA worked to build an international infrastructure of linked national centers designed to facilitate the development of a transnational laboratory animal science community. The transfer of expertise from Norway to Thailand is illustrative of how ICLA enabled expertise to be developed and communicated across this network regardless of the relative strength of the biomedical sciences at the point of origin. Indeed, enabling countries with modest or developing biomedical communities to communicate directly was a necessary strategy to foster the development of appropriate local laboratory animal provision. This work, however, was not merely engaged in building an international infrastructure for laboratory animal science. ICLA’s activities were directed toward shaping the material cultures of animal dependent science. The extensive surveys were not just a benign act of measurement. Rather, they operated to constitute that which they purportedly quantified by mapping the transnational material spaces of science (laboratories and animal houses) and bringing into existence transitional populations of laboratory animals. By bringing populations of animals into existence that were defined by science as opposed to geographic origin, ICLA was creating new objects to be shaped as well as a perceived need for standards, expertise, and technologies to perform the shaping.

## Standardizing Laboratory Animals – Building Transnational Bodies

As a consequence of ICLA’s work, laboratory animals gained new identities, becoming globally transacted objects existing unhindered by their local (national) sites of origin. By extension, local problems that had long plagued laboratory animal users were recast in their significance and scope as problems of international significance that could only be answered by entering into transnational dialogues. At the Laboratory Animal Division of the NIPH in Norway in 1960, the newly appointed veterinarian, Stian Erichsen, began to reinterpret longstanding problems with laboratory animal health, disease, and economies of production through an international lens. In one case, an outbreak of ectromelia virus (commonly called “mouse pox”), the symptoms of which were skin lesions, gangrene and death, was blamed on the introduction of mouse stock from a Danish commercial breeder (Erichsen 1961a). The Danish animals were found to tolerate ectromelia whereas their “healthier” Norwegian cousins, never having encountered the virus, could not. In the 1950s, the relationship between geographic background, pathogenic history, and laboratory animal disease epidemics formed an important area of research for early laboratory animal science. At the time, little was known about laboratory animal epidemiology. Causative agents of disease were largely unidentified, making their detection impossible unless (and until) they revealed themselves through obvious symptoms or unexplained deaths. The production of healthy, infection free, laboratory animals became one of the most widely held definitions of “quality,” both with reference to economics of animal provision and reliability of animals as experimental tools. The latter because the capacity of animals to tolerate latent infections was dependent upon the balance of their physiological and environmental state, a balance that could be quickly disrupted by laboratory interventions. The outbreak of disease during experiments could undermine results by making the procedure impossible to complete or more difficult to detect, and could be interpreted as a consequence of the experiment, thereby producing misleading results. In either case, *latent infection* was a factor that had to be controlled if experimental results were to be credible and replicable.

The complexity of infection was mobilized to justify the need for original research in the field of laboratory animal science and medicine (Lane-Petter 1956). In terms of health, therefore, improving quality not only required the prevention of disease but also required methods to produce, maintain, and define “healthy” laboratory animals (Tuffery 1962). Definition, in turn, necessitated knowledge of what was to be controlled against, which in turn demanded some means to render visible then unknown laboratory animal pathogens. Until substantial progress was made in what was, essentially, a new field of laboratory animal epidemiology, alternative means of managing animal health had to be employed. These techniques were structured spatially, essentially adapting the long tradition of quarantine to create new biomedical geographies for the animal house and laboratory.

**Fig 1:**
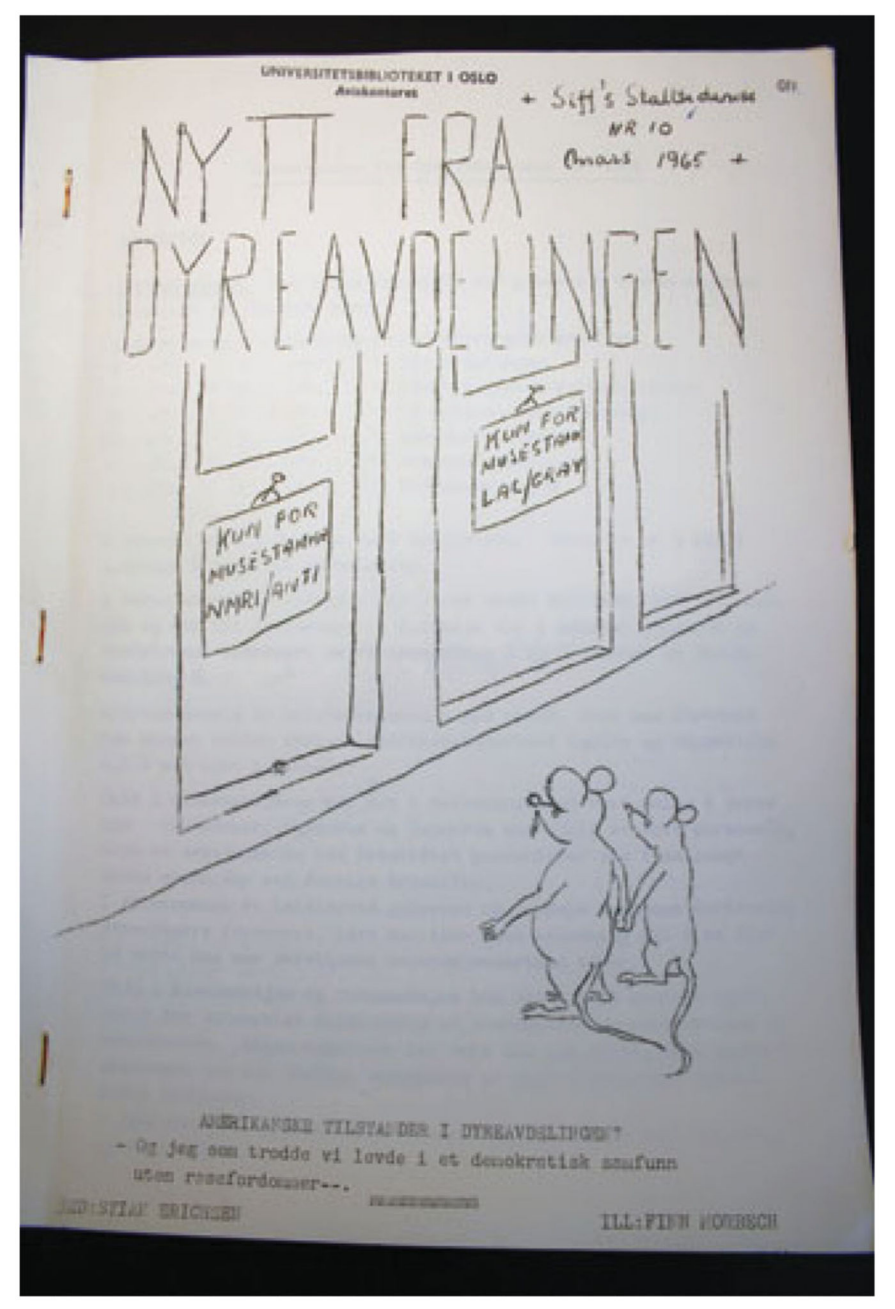
“And I thought we lived in a democratic society without racial prejudices” (Source: SIFF Stalltidende, No. 10, March 1965).

Erichsen responded to the 1960 epizootic outbreak by introducing a series of new spatially orientated preventative practices designed to prevent future epidemics. All new animals entering the animal house were quarantined for a set period before being introduced to existing stocks. Further, accurate systems of tracking and management were introduced to ensure that animals originating from different localities were maintained in isolation (Erichsen 1961a). These regimes were designed to overcome the problem of local specificity making laboratory animals transferable and available to all, and were crucial in the re-configuration of laboratory animals as transnational bodies, divorced from national backgrounds. However, these same practices also operated to reinforce locality as fundamental to the identity and proper use of a given animal stock. This irony was not lost to Erichsen, as demonstrated by the front cover of a 1965 in-house animal division newsletter depicting two Nordic mice looking up at two doors clearly marked “NMRI/ANTI mice only” (a USA mouse strain) and the other “LAC/GRAY mice only” (a UK mouse strain). One mouse says to the other “American only animal houses at the Animal Department? And I thought we lived in a democratic society without racial prejudices” (see fig. 1).

Whilst practices such as these worked to overcome arbitrary associations between nation of origin and the laboratory animal body, ensuring there would be no more “English Guinea Pigs” or “Swiss Mice,” they nevertheless re-inscribed local laboratory animal identity about the site of animal production (thus the NMRI mouse gained identity from having originated at the US Naval Medical Research Institute). In order to truly construct the laboratory animal as a transnational body, the link between pathogenic and geographic history had to be broken.

**Fig 2:**
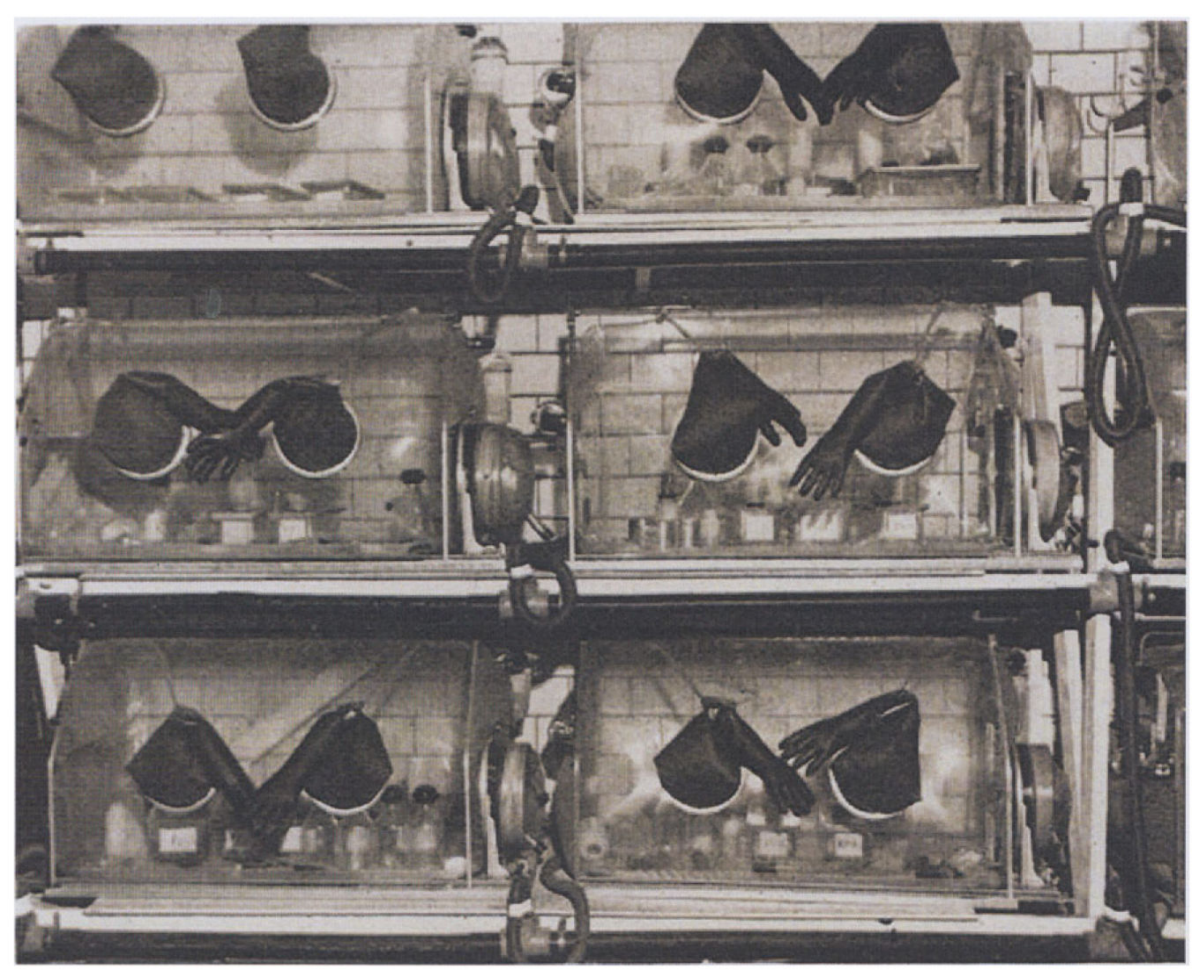
Trexler Plastic Isolators (Source: Trexler 1959, 32).

In 1961, ICLA coordinated a symposium addressing just this issue, the original title of which, “The Elimination of Infectious Diseases from Laboratory Animal Colonies,” was indicative of the intent and hopes of early laboratory animal science.^28^ The agenda was to explore methods of producing laboratory animals that were capable of isolating animal bodies from local microbial environments specific to the place of production. Over five days, all manner of issues relating to the quality of animals were discussed, focusing on species, specific regimes of disease identification, prevention, and monitoring. The central event, however, was the exhibition of newly developed techniques and technologies for “germ free animal” production (ICLA 1961, 6). Speakers included Philip C. Texler, of the Lobund Institute (University of Notre Dame, Indiana), a leading pioneer of germ-free techniques, alongside representatives from the National Institutes of Health (USA) and M. Sabourdy of the French Centre de Selection des Animaux de Laboratoire (Gif-sur-Yvette, France). By the early 1960s, however, a cheap, simple to construct, easily adaptable isolator had been developed made of durable plastic by Philip C. Texler (Trexler 1959). It was this technology that ICLA popularized as a means to make microbially defined laboratory animals available to all (see fig. 2). Such animals, being “free of specified micro-organisms and parasites,” were defined as Specific Pathogen Free or SPF (ICLA 1964, iii).

ICLA was interested in popularizing the technology of germ-free animal production as it could be adapted to breed animals of known microbial loads, an important technique that would make it possible to divorce the identity of animals from the arbitrary link to local site of production.

Erichsen was keen to introduce these techniques into Norway as, if widely established, SPF colonies promised to eradicate the problem of latent infection amongst Norwegian laboratory animal stocks. However, despite innovations such as cheap plastic isolator technology, these techniques remained economically challenging as SPF-derived animals were substantially more expensive. As Erichsen explained: “Laboratory animals cost money as other goods. The prices the users have to pay for the animals are of course dependent on many factors, for instance the price of production (feeding, cages, work load, housing etc). The organisation of the work and the size of the production unit will play a part, but so will certain demands for quality.”^29^ Erichsen explained that moving to the SPF standard – though initially expensive in terms of upgrading, animal houses, and cost per animal – would in the long term bring economic savings. Due to the higher standard of health, fewer animals would be required to gain the same level of statistical significance in a given experimental procedure. This in turn would allow animal houses to reduce their stocks without undermining their ability to meet the demands of researchers. Many remained skeptical of the economic case for a wholesale switchover to SPF standard. Most researchers were slow to recognize the value of SPF until they witnessed its utility in practice. Consequently, Erichsen adopted a principle to “standardize where it is possible,” by which he meant a program to align Norwegian science with the trajectory of ICLA whilst working with its limits in order to meet its needs. Having upgraded the animal house at the NIPH, Erichsen began transposing the animal stocks to the SPF standard. Unable to initiate an indigenous Norwegian breeding program, he instead opted to refashion the Laboratory Animal Division as *an importer and distribution hub* for SPF animals sourced from the US commercial producer Charles River (which had opened its first European facility in France the previous year) and from a producer in Denmark.^30^ This approach was designed to facilitate Norwegian access to SPF animals whilst enabling users to focus their limited resources on upgrading animal house facilities and learning new husbandry skills necessary to maintain these animals.^31^ Erichsen’s strategy thus mediated between local needs and transnational standards, allowing Norway to participate in the international development of laboratory animal science in such a way that satisfied the highly localized circumstances and needs of the Norwegian biomedical sciences. Doing so enabled access to laboratory animals of known backgrounds, defined entirely by their pathogenic loads as opposed to site of production.

Without a transnational space and infrastructure Norwegian laboratory animal science might not have been a successful project. In many ways, it actually wasn’t a success; biomedical science in Norway is crucially dependent on importing animals as production of animals is not of high priority, mostly due to the cost and time-consuming task of producing laboratory animals and due to more experience and suitable infrastructures in other countries such as Sweden and Denmark. The dependency on other countries to ensure sufficient supply of laboratory animals is a fragile contract as even though animals were smoothed into transferable objects, national politics was always posing a threat to the system. Erichsen experienced this fragility in 1967 when animal rights activists in the UK had managed to push a ban on export of laboratory animals to other countries by pointing to the fact that UK regulations were not transgressing borders; how could English citizens know that animals were handled with care in other countries? Erichsen rushed the Ministry of Social Affairs to respond to the British government by stressing how this ban would seriously threaten national biomedicine as the UK was its central provider of SPF animals.^32^ Thus even though standardizing the bodies of animals in order to transcend political and scientific differences between nations, the origins of the animals were made central by factors such as animal welfare concerns and national responsibilities.

## Conclusion

Laboratory animal science and medicine was structured around national centers which, in many ways, acted as hubs creating, distributing, and channeling new knowledge, as well as the means, materials, and technologies, into international networks co-ordinated by ICLA. As a field, the active centers of Laboratory Animal Science and Medicine were always grounded in local, national concerns. It is notable, in this perspective, that ICLA itself was not a “Center” but a “Committee,” working to facilitate the circulation of laboratory animal science by building international networks. Thus, national practice and the building of transnational standards were locked within a dynamic process of mutual constitution, which, channeled through national centers, was informed by activities and transactions on the international stage as mediated by ICLA. This process can be viewed as an example of what has been termed “world scientization,” a symptom of which is the growth of “the putatively disinterested experts who roam the nation and the world, providing advice on how individuals, organizations, and nation-states, may achieve development goals (Drori et al. 2003, 295). For example, ICLA’s sponsoring of “Visiting Experts,” such as Erichsen’s work in Thailand during the 1970s, fits this model. Visiting Experts were the principle procedure by which the means, materials, and technologies traveled from one locality to another. The process of international movement and re-instantiation was always transformative, the friction emerging from national difference proving creative in the sense described by Tsing. It is the friction from national difference that allows “universals,” such as scientific standards, to become active, effective, and engaging. For Tsing, therefore, standards would be less immutable universals than “sticky engagements” continually being transformed by their travels (Tsing 2005, 5–8).

The ability to produce laboratory animals with known pathogenic status, so-called SPF animals, realized the ideal of the laboratory animal as a transnational body, working across national culture and local site of production, bringing into being an animal defined only by scientific standards. Moreover, the SPF process proved to be a durable standard, mediating and embodying the tensions between local specificity and transnational scientific standards, because they conformed to an agreed nomenclature (and therefore met the criteria of being “universally” defined) whilst simultaneously being endlessly reconfigurable depending on the pathogenic definition (thus meeting the local contingencies of a given laboratory, research trajectory, or experimental practice). The creation of this form of life, born of and for the laboratory, was made possible through the frictions emerging from national differences. Techniques such as SPF animal production worked to transform the laboratory animal into a transnational body – a materially embodied interface connecting local situated practice with the idealized transnational standards of science.

Thus, the means, materials, and technologies of laboratory animal science were always locally determined (as for instance in Erichsen’s strategy to “standardize where it is possible”); they worked as “sticky engagements.” Indeed, we would argue that the transnational was an outcome of local practices and technical arrangements which, though geographically dispersed, worked toward the same ends. From this perspective, the historical formation of laboratory animal science and medicine has to be understood as emerging, literally and figuratively, from multiple, geographically dispersed, centers (MacLeod 2000). The Norwegian NIPH worked productively through the frictions of national difference to produce new ways of working with laboratory animals that met local needs whilst remaining mobile and thus in transnational creative dialogue with other centers of laboratory animal science and medicine across the world.

## Footnotes

1 Put another way, the work of laboratory animal science focused on developing laboratory animals of all species, together with regimes of maintenance and care. The outlook was predominantly species orientated as opposed to focusing on creating specific models for specific research agendas.

2 In 1952, the US National Academy of Sciences organized a conference, chaired by Clarence Cook Little of the Jackson Memorial Laboratory, to consider the best means of nationally organizing animal procurement for the biomedical sciences, which subsequently became the Institute of Laboratory Animal Resources (ILAR) “to act as a clearing house for the collection, correlation, evaluation and dissemination” of information relating to laboratory animals (Herrlein 1954, v).This was not the first time Little had been asked to explore the need for a national framework of animal provision. Working with the geneticist L. C. Dunn between 1928 and 1932, Little had tried and failed to establish the need for a centralized system to preserve and provide animals for experimental use. The project floundered due to an inability to agree on whether centralization was the best means to organize science (as some feared it might curtail researchers’ autonomy) and, more critically, at the time few researchers saw animal provision as a pressing problem (Rader 2002). A similar process occurred in the Netherlands, where the Centraal Proefdieren Bedrijf (TNO) was established in 1949, and in France resulting in the establishment of the Centre de Sélection des Animaux de Laboratoire (Gif-sur-Yvette) in 1952.

3 Norway had since the Second World War actively developed international relations to enable growth in national industry by promoting the development of international regulations and systems of exchange (see Asdal 2011).

4 CIOMS was established by the World Health Organization (WHO) and UNESCO in 1949 to promote the global development of the biomedical sciences.

5 “Meeting of Consultants on International Co-operation in the field of Laboratory Animals, 3-4 December 1956”, File NS/BIOCEL/13; WS/037.18, UNESCO Archives, 7 place de Fontenoy, 75352 Paris 07 SP, France (hereafter UA).

6 In recent years historical scholarship has probed the complex overlapping meanings of the terms “national,” “international,” “world,” “global,” and “transnational” (Iriye and Saunier 2009). Here we understand “international” to retain the nation as a unit, “global” to transcend the nation roughly encompassing the “world,” and “transnational” to transcend the nation without implying a global scope. For a historical analysis of the modern geopolitics of health beyond the nation state, see Bashford (2006) and for a critical discussion of concepts of international, world and global health, see (Keane 1998; Brown et al. 2006).

7 “Meeting of Consultants on International Co-operation in the field of Laboratory Animals, 3–4 December 1956”, File NS/BIOCEL/13; WS/037.18, UN.

8 Historiography on the relationship of local to global (particularly in relation to postcolonial studies) is vast. Anderson (2002) provides a useful introduction to the intellectual stakes of such questions for the study of science.

9 For the critical role of standards as drivers of globalization and “world scientization,” see Drori et al. 2003.

10 Olav Torgersen and Ernst Wulff Rasmussen “Vedlegg til søknad R. 3 1954/55 [Attachment to Application R. 3 1954/55]”, 1953, Archives of the Norwegian Research Council, Box 113, National Archives, B. 4031 Ullevål Stadion, 0806, Oslo, Norway (hereafter ANRC).

11 The Norwegian Research Council (NRC) was established in 1949.

12 Ragnar Nicolaysen “Referat fra møte den 1. Oktober 1955 i NAVF’s komite for dyrestall for forsøksdyr [Minutes from NRC Committee on Laboratory Animal Breeding Station]”, 1st October 1955, Archives of the Norwegian Research Council, Box 113. ANRC.

13 Historians have shown how the immediate postwar years witnessed the transformation of medicine through the scaling up of laboratory-based biomedicine (Cunningham 1992; Keating & Cambrosio 2003; Gaudilliere & Lowy 1998). Margaret Lock and Vinh-Kim Nguyen have pointed out that biomedicine can be understood as technologies of bodily governance. Building and extending on Michel Foucault’s notion of biopower, Lock and Nguyen show how biomedical technologies are not only technologies found in clinics and hospitals such as X-ray machines or CT scans, but are highly efficient governing technologies in disseminating biomedicine in lives and worlds. That is, they work as technologies for instance in deploying representative samplings and statistical assessments that target individual bodies as well as communities, nations, and at times the whole world (Lock and Nguyen 2010, 23–24). Thus, in this view, biomedicine is an assemblage of methods, technologies, and infrastructures that are aimed at governing bodies and societies by effective means.

14 For instance, Evang routinely sent his staff to be trained in healthcare administration at Johns Hopkins University (Ringen 1990).

15 In the press release announcing the planned construction of the Virus laboratory at the NIPH, director of health Karl Evang listed fourteen different epidemic diseases that plagued or could potentially plague the Norwegian population if measures were not taken in preventative medicine. Polio was regarded as particularly threatening, and in 1956 a free national vaccination program was initiated in Norway, administered by the NIPH, targeting polio in particular. The Nordic countries also cooperated to import the Salk vaccine through the Nordic committee for polio (Karl Evang, “P.M. Vedr, Viruslaboratoriet” [Note on the Virus Laboratory], 23. November 1954. Archives for the Ministry of Social Affairs. Directorate of health. Office of hygiene H5. Box 20, ANRC).

16 Ragnar Nicolaysen “Referat fra møte den 1. Oktober 1955 i NAVF’s komite for dyrestall for forsøksdyr [Minutes from NRC Committee on Laboratory Animal Breeding Station]”, 1st October 1955, Box 113. ANRC.

17 NRC Working group “Nr. 93/58: Innstilling fra NAVFs utvalg for reisning av avlsstasjon for forsøksdyr, 11. desember 1958” [Final report from the NRC Working group on a centralized breeding station for laboratory animal science 11 December 1958] Archives of the Norwegian Research Council, Box 115, ANRC.

18 See Asdal 2008 for a study on the controversy of the laboratory and laboratory animals around 1900.

19 Christian Lerche, “Avlsstasjon for laboratoriedyr på Bygdøy Kongsgård” [Laboratory Animal Breeding Station at Bygdøy Royal Farm], 1960, Box 781, ANRC; Helge Stormorken “Avlsstasjon for laboratoriedyr på Bygdøy Kongsgård” [Laboratory Animal Breeding Station at Bygdøy Royal Farm], 1960, Archives of the Norwegian Research Council Box781, ANRC.

20 Helge Stormorken, Letter to NRC, 17^th^ October 1960, Archives of the Norwegian Research Council, Box 781. ANRC.

21 Expectations were that the farm would initially build up to producing 40,000 mice, 15,000 rats, 3,000 guinea pigs, and 2,000 rabbits annually before developing dog and cat breeding programs (ICLA 1957, 16).

22 Meeting of Consultants on International Co-operation in the field of Laboratory Animals, 3–4th December 1956”, p. 6, File NS/BIOCEL/13; WS/037.18, UA.

23 A legacy of this is the Scandinavian Society for Laboratory Animal Science (Scand-LAS), established in 1970 as a mechanism to develop Scandinavian laboratory animal science and partly modelled on ICLA.

24 For instance, in 1957 the Zentralinstitut für Versuchstierzucht was formed in the Federal Republic of Germany. In 1958 India established the Laboratory Animal Information Service; Belgium set up the Comite National Provisoire sur les Animaux de Laboratoire in 1958; Australia, a Laboratory Animal Centre in 1959; and Italy the Centro di Informaziomi sulla Produzione degli Animali di laboratorio the same year (Bleby 1967).

25 Christian Lerche “Overlevering av det nye viruslaboratorium ved Statens Institutt for Folkehelse” [The new Virus Laboratory at the National Institute of Public Health], 27th April 1960, Box H5, Office for Hygiene, Directorate of Health, Department of Social Affairs, National Archives, Oslo.

26 Helge Stormorken “Forsøksdyrproblemet i Norge” [The problem with laboratory animals in Norway], 1968, Archives of the Norwegian Research Council, Box 781. ANRC.

27 For Erichsen’s co-ordination of a scientific response to Council of Europe Recommendation 621 (1971), see “IAAPEA & ICLA”, File PP/WDP/E/5, Sir William Drummond Macdonald Paton Collection, Archives and Manuscripts, Wellcome Library for the History of Medicine, 183 Euston Road, London, UK.

28 Held in Liblice and Smolenice, then cities of the Communist Socialist Republic of Czechoslovakia, and supported by UNESCO and the National Institutes of Health of the USA, this meeting is equally illustrative of the way in which laboratory animal science operated to transcend the geopolitical division of Democratic West and Communist East as it was played out in the 1950s and 1960s.The USSR was an active member of ICLA, for example, albeit one occasionally hampered by inability to attend meetings or pay membership. On the invitation of the Academy of Medical Sciences (Moscow) Lane-Petter visited a number of Soviet facilities between 19 January and 1 February, 1960, noting in particular that Russian laboratory animal science was particularly advanced in the production and husbandry of nonhuman primates (ICLA 1961).

29 “Ny musestamme” [New mouse strain], Stian Erichsen, 1967. Nytt fra Dyreavdelingen [News from the Animal Division], no. 15. National Library, Oslo.

30 Gamle Bomholdt gård (Old Bomholdt farm) was the name of the Danish breeder. Erichsen was involved in planning the arrangement of changing Gamle Bomholdt gård’s breeding program from producing conventional animals to animals of SPF quality in the late 1960s. “Mus,” [Mouse], Stian Erichsen, 1967. *Nytt fra Dyreavdelingen* [News from the Animal Division], no. 16. National Library Oslo.

31 “Bør vi også produsere annenklasses dyr?” [Should we also produce second class animals], Stian Erichsen, October 1972. *Nytt fra Dyreavdelingen* [News from the Animal Division], no. 30. National Library Oslo.

32 Interestingly, Erichsen suggested in the note to the Ministry that to ensure a steady supply from Britain could take a bilateral agreement that did not include the international community of researchers. A bilateral agreement would have a better chance to succeed in this particular case than to develop an international licensing system. “Note fra den britiske ambassade angående bestemmelser vedrørende forsøk med dyr” [Note from the British embassy regarding animal experimentation], Stian Erichsen 6^th^ June 1967, NIPH Administrative Archive, Box 53, Folder 3.10.09 *Dyrehold* [Animal husbandry]. National Archives, B. 4031 Ullevål Stadion 0806, Oslo, Norway.
